# The Distinct Structural Propensities of Poly-C, A, and U Single-Stranded RNA

**DOI:** 10.1021/acs.biomac.6c00716

**Published:** 2026-08-05

**Authors:** Tong Wang, Weiwei He, Scout Fronhofer, Serdal Kirmizialtin, Lois Pollack

**Affiliations:** † School of Applied and Engineering Physics, 5922 Cornell University, Ithaca, New York 14853, United States; ‡ Chemistry Program, Science Division, 167632 New York University Abu Dhabi, Abu Dhabi 129188, UAE; § Department of Chemistry, New York University, New York, New York 10003, United States

## Abstract

Homopolymeric single-stranded RNAs (ssRNAs) are common biological motifs, yet their sequence-dependent solution structures remain incompletely defined. Particularly, rC30 and rA30 have not been characterized with atomic detail. Using optimized force fields, we integrate small-angle X-ray scattering (SAXS) with SAXS-driven molecular dynamics to generate and refine conformational ensembles for thirty-nucleotide-long strands of poly­(rA), poly­(rC), and poly­(rU) (rA30, rC30, and rU30) in identical buffers. Scattering profiles are computed from refined MD-generated ensembles and accurately reproduce the SAXS measurements. Properties of these refined ensembles are further validated by circular dichroism (CD) and UV melting. Clear sequence-dependent order emerges: rA30 is the most compact and helical, rU30 is largely coil-like, and rC30 falls in between. Together, these cross-validated ensembles define distinct conformational propensities of ssRNA homopolymers, specifically highlighting poly­(rC) as a unique, moderately structured, yet highly heterogeneous ssRNA. These findings may have implications for nucleotide-specific macromolecular recognition.

## Graphical abstract

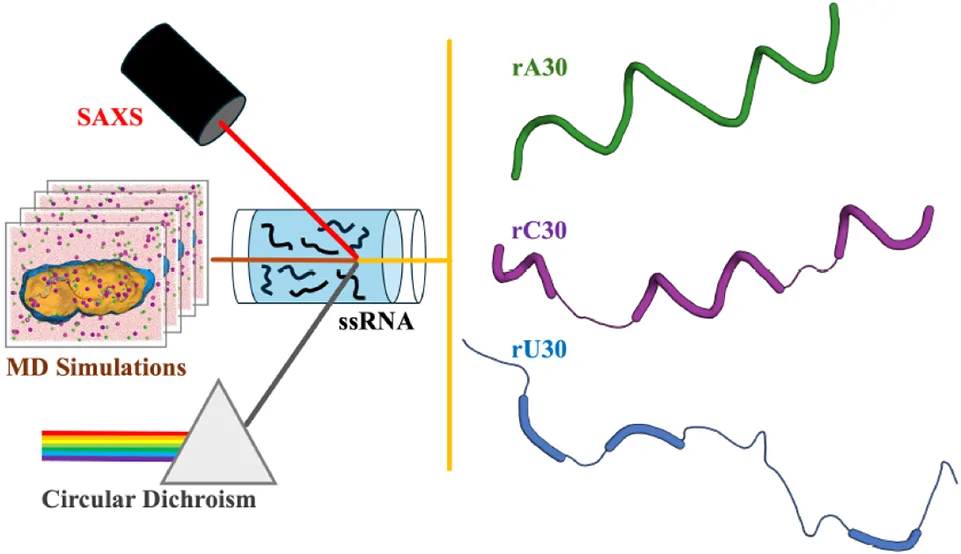

## Introduction

RNA assumes a variety of structures that facilitate its many functions. Single-stranded regions in RNAs (ssRNAs) are abundant, both as nonbase paired components of larger molecules or as structural motifs involved in biological interactions. Notably, homopolymer tracts of all four RNA nucleotides (adenine (A), uracil (U), cytosine (C), and guanine (G)) with up to hundreds of repeats are observed in nature.^1^ With the exception of poly­(rG), which readily assembles into G-quadruplexes^2^ tandem units of A, U, and C remain single-stranded (predominantly nonbase paired) in solution. Despite their flexible nature, these ssRNAs can display remarkable specificity when selecting binding partners.^3,4^ Poly­(rA) tails, found in mRNA and RNA viruses, are involved in the stabilization and export of mRNA from the cell nucleus, as well as signaling for mRNA degradation.^5^ In some viral RNAs, poly­(rU) tracts are recognized by the cell as a signal to attack the virus, triggering an immune response.^6,7^ The interactions of poly­(rC) binding proteins with C-rich RNAs are connected to regulatory roles in transcription and translation.^4,8^

Recent coupled experimental and simulation studies have begun to reveal the conformers that populate the structural ensembles of these single-stranded RNA homopolymers. The first comprehensive study deriving and comparing the conformational ensembles of rA30 with rU30 provided evidence for conformational differences between the two, as indicated by numerous measurements; helical (A-form-like) conformations exist in poly­(rA), while poly­(rU) populates less ordered, coil-like states.^9^ In this study, interpretation of experimental data relied on refinements of simple polymer models derived from chain-building algorithms constructed from dinucleotide models. More sophisticated chain-building^10^ or coarse-grain^11^ algorithms have recently been applied to model these ssRNAs, reaching similar conclusions. Molecular dynamics (MD) simulations coupled with both small-angle X-ray scattering (SAXS) and nuclear magnetic resonance (NMR) have been applied to study shorter (hexamer) strands.^12^ More recently, all-atom MD simulations coupled with SAXS and Förster resonance energy transfer (FRET) data revealed distinct structural differences between rU30 and a mixed-sequence RNA rich in A and C.^13^ Interestingly, this latter study suggests that C-rich regions of single-stranded RNA form structures with helical properties that differ from A-rich regions. Since most prior studies of homopolymeric ssRNA structures have focused on articulating the differences between ensembles of poly­(rA) and poly­(rU), this observation motivates the need to explore the structural ensembles of poly­(rC) homopolymers.

The existence of helical structures in poly­(rC) has long been recognized, dating back to early fiber diffraction and relaxation kinetics studies, which suggested that it adopts right-handed, stacked single-strand helices at neutral pH.^14,15^ Though left-handed structures have also been detected,^16^ short RNA chains, in recent NMR measurements, find right-handed propensities for poly­(rC).^17^ At the same time, temperature-jump experiments reported helix–coil interconversion and solvent-dependent stacking, finding faster transitions in poly­(rC) than in poly­(rA).^18^ Single-molecule force spectroscopy provided evidence for alternating stacked-helical and coil domains in homopolymers of poly­(rC) and poly­(rA), enabling a sequence-dependent mechanical response, with weaker stacking in the former relative to the latter.^19,20^ Single-molecule techniques have also demonstrated the sequence dependence of chain dynamics in single-stranded nucleic acids, showing that the reconfiguration time for poly­(rC) is intermediate between the other homopolymers.^21^

Taken together, the prior work on poly­(rC) suggests that it is structured in a way that is distinct from the other homopolymers. Here, we characterize the structures of poly­(rC) using a strategy that integrates MD simulations with multiple experimental methods. To facilitate a direct comparison with poly­(rA) and poly­(rU), those polymers were investigated as well. Structural ensembles derived from SAXS-driven MD simulations are used to interpret circular dichroism (CD) and UV melting experiments for poly­(rA), poly­(rC), and poly­(rU) oligomers measured under identical ionic conditions. In this assessment, poly­(rC) emerges as intermediate in compaction and helicity, with short stacked segments and helical periodicity that distinguish it from poly­(rA) and poly­(rU). Notably, poly­(rC) exhibits both helical order and high structural heterogeneity. These results provide a comprehensive comparison for homopolymeric ssRNA under matched conditions. The distinct structural characteristics of poly­(rC) may facilitate the recognition of C-rich strands by proteins.

## Experimental Section

### Small-Angle X-ray Scattering (SAXS) on Single-Stranded RNA

Lyophilized RNA was purchased from Dharmacon (Horizon Discovery, Waterbeach, UK), reconstituted, and exchanged in 100 mM NaCl, 1 mM MOPS (titrated with 10 M NaOH to pH 7.07), 20 μM EDTA buffer using 3k molecular weight cut off 0.5 mL centrifugal filters. Before measurements, RNA was annealed by heating the sample to 90 °C for 3 min and snap cooling to 4 °C for 20 min, then stored on ice until measurements were performed. RNA concentrations were measured on a Nanodrop (ThermoFisher) after diluting the sample 20× and 100×, averaging three acquisitions and verifying that the two dilutions gave similar values. Before SAXS measurements, samples were centrifuged at 14000 rpm for 2 min.

SAXS measurements on rC30 were performed at the 7A-1 BioSAXS/High-pressure SAXS beamline at the Cornell High Energy Synchrotron Source, with the sample at ambient temperature. Images were radially integrated using BioXTAS RAW such that the scattering intensity I­(q) is plotted as a function of scattering vector $q=\frac{4\pi \sin\left(\theta \right)}{\lambda }$ , where λ is the wavelength of incident X-ray radiation and 2θ is the scattering angle. 10–100 × 1 s exposures were acquired and averaged, confirming that no radiation damage occurred across traces by monitoring changes in low q. SAXS data on rU30 and rA30 in 100 mM NaCl are derived from Plumridge et al.^9^ To correct for concentration-dependent RNA interparticle repulsion effects at low q, SAXS data were acquired at multiple concentrations, and low q scattering (*q* < 0.05 Å^–1^) was linearly extrapolated to the zero concentration limit.

Radii of gyration were determined from the SAXS data using Guinier analysis, fitting the scattering to the equation

1 $$\log I\left(q\right)=-\frac{R_{g}^{2}}{3}q^{2}+\log I\left(0\right)$$

Plots of log­(*I*(*q*)) vs *q* ^2^ were fit to obtain *R*_*g*_ (from the slope) and I(0) (from the intercept).

### Circular Dichroism

ssRNA for CD was prepared the same way as for SAXS, and diluted to 1–2 μM. Spectra were acquired using a BioLogic MOS 450 in CD mode and Xe–Hg lamp illumination. Monochromator scanning from 220 to 300 nm, where RNA helical features are pronounced, was done with a step size of 0.5 nm and an acquisition period of 1 s. Fifteen scans were collected and averaged.

### Single-Stranded UV–Vis Melting

ssRNA melting curves were collected on a Agilent Cary 3500 UV–vis spectrophotometer, on ssRNA that was prepared the same way as for SAXS but diluted to 1–2 μM. The sample was heated from room temperature up to 80 °C, then cooled to 4 °C in 1°/s intervals. To prevent condensation buildup on the sample cell at lower temperatures, *N*_2_ gas flow into the sample chamber was maintained for the duration of the measurement.

### Computational Modeling of ssRNA Ensembles

We conducted a two-step simulation protocol to generate conformational ensembles of ssRNAs. Full methodological details are provided in the Supporting Information, and a concise summary is provided below.

Each RNA molecule was solvated in a 12 × 12 × 16 nm^3^ simulation box with explicit water and ions, using conditions matching experimental setups. All-atom MD simulations were performed using GROMACS 2018.5 ^22^ with the HB-CUFIX force field, which incorporates hydrogen bonding and base stacking corrections^23^ for RNA and has proved to reproduce RNA dynamics consistent with experimental observations.^24^ A 200 ns brute-force MD simulation at 300 K was used to explore the initial RNA conformational space. Sampled structures were clustered by RMSD, and four conformers representing the most populated clusters were selected as replicas for multireplica SAXS-driven MD simulations,^13^ which incorporated a hybrid potential energy function of the form *E*_hybrid_ = *E*_FF_ + *E*_SAXS_. Here, the MD force field energy (*E*_FF_) was combined with a time-dependent SAXS restraint term (*E*_SAXS_) applied independently to each replica, as described previously:^13,25,26^

2 $$E_{\mathrm{SAXS}}\left(R_{1},\;...,\;R_{N},\;t\right)=\alpha \left(t\right)k_{c}N^{\Omega }\frac{k_{B}T}{n_{q}}\overset{n_{q}}{\underset{i=1}{\sum }}\frac{{[{\mathrm{I̅}}_{comp}(q_{i},\;t)-I_{\exp}(q_{i})]}^{2}}{\sigma^{2}\left(q_{i}\right)}$$

where *I̅_c__omp_* (*q_i_*, *t*) is the replica-averaged computed intensity, and *I*_exp_(*q_i_*) is the experimental intensity at scattering vector *q*_*i*_. The weight factor *k*_*c*_ *N* ^Ω^ scales the SAXS potential relative to the force field, and α­(*t*) gradually increases SAXS coupling during simulation. The error term σ^2^(*q*_*i*_) includes contributions from experimental, statistical, and buffer uncertainties: $\sigma^{2}\left(q_{i}\right)=\sigma_{\exp}^{2}\left(q_{i}\right)+\sigma_{\mathrm{comp}}^{2}\left(q_{i}\right)+\sigma_{\mathrm{buffer}}^{2}\left(q_{i}\right)$ . The last 100 ns of each replica where χ^2^ < 1.0 were used to construct final conformational pools for each sequence. Details on SAXS-MD simulation setup are provided in the Supporting Information. The structural descriptors, including radius of gyration, base stacking, and orientational correlation functions, were extracted from simulations using PLUMED 2.6.1 ^27^ and metrics computed by x3dna-dssr.^28,29^

### Computing CD from MD Simulations

We employed the DichroCalc program^30^ to compute theoretical CD spectra. DichroCalc implements the matrix method, which models exciton coupling between chromophores and requires a set of parameters—such as transition energies and dipole moments—that can be derived either empirically or *ab initio*.^30^ In our study, we used the *ab initio* parameter set provided by the program, applied backbone only transitions (set to two), and specified the spectral range of 220–305 nm, while keeping all other parameters at their default values. Based on the settings, CD spectra of 2000 conformers were computed and averaged. The original adenine (A), cytosine (C), and uracil (U) excitation-parameter files used in DichroCalc CD calculations are provided in Supporting Information.

### Helical Handedness Analysis of ssRNA

For each ssRNA conformer, we enumerate all consecutive residue spans of length ≥ *L*_min_ (*L*_min_ = 3 in this study) and classify each span’s handedness. For a candidate span $\{r_{i}{\}}_{i=1}^{k}$ , we construct a local helical frame by (i) centering the coordinates, (ii) estimating the principal axis and orienting the segment from the first to the last phosphate (defining **ẑ**), and (iii) defining an orthonormal basis (**x̂**, **ŷ**, **ẑ**) with **x̂** in the plane orthogonal to **ẑ**. Each P atom is projected to (*x*_*i*_, *y*_*i*_, *z*_*i*_) in this frame, and the in-plane azimuthal angle is computed as θ_*i*_ = atan2­(*y*_*i*_, *x*_*i*_) along the span.

The handedness is determined by the parameter *s* with *s* > 0 right and *s* < 0 left-handed. To compute *s* we quantify twist per rise by the least-squares slope of θ versus *z*:

$$s=\frac{\overset{k}{\underset{i=1}{\sum }}\left(z_{i}-\mathrm{z̅}\right)\left(\theta_{i}-\mathrm{\theta ̅}\right)}{\overset{k}{\underset{i=1}{\sum }}{\left(z_{i}-\mathrm{z̅}\right)}^{2}},,\mathrm{z̅}=\frac{1}{k}\underset{i}{\sum }z_{i},,\mathrm{\theta ̅}=\frac{1}{k}\underset{i}{\sum }\theta_{i}$$

We apply a threshold cutoff to the mean per-step turn, ⟨Δθ⟩, assigning handedness only to spans with clear rotation (|⟨Δθ⟩| ≥ δ, δ = 50° here). The resulting handedness trends are robust to threshold choices from 20° to 60° (Figure S8).

## Results and Discussion

### Homopolymeric ssRNA Shows Sequence-Specific Experimental Differences

As a first step in investigating differences between poly­(rC), poly­(rA), and poly­(rU), we characterized the conformational distributions of the ssRNA homopolymers rA30, rC30, and rU30 in solution. We selected 30-nucleotide chains for this study because they populate a regime in which polymer-like behavior, including Flory-like scaling, is observed, while the scaling exponents remain sequence-dependent.^13^ Thus, sequence-dependent effects are visible at this length. SAXS measurements of these three RNAs revealed distinct scattering profiles acquired under the same salt conditions (Figure 1A), suggesting that nucleotide identity impacts overall size and shape. Both the radii of gyration and subtle differences in the high angle scattering indicate sequence-dependent differences in molecular size and conformation. The radius of gyration of rU30 is nearly 25% (5 Å) larger than that of rA30, with rC30 intermediate in size (Figure 1B). CD spectra of the three sequences (Figure 1C) show unique profiles, suggesting distinct helical order. rU30 shows a weaker helical signal compared to rA30, which displays a strong peak at around 260 nm, similar to that of an A-form helix.^31,32^ The CD profile of rC30 is distinct from both, showing strong, but shifted peaks, including a shift toward the higher wavelengths associated with B form DNA.^33^

**Figure 1:**
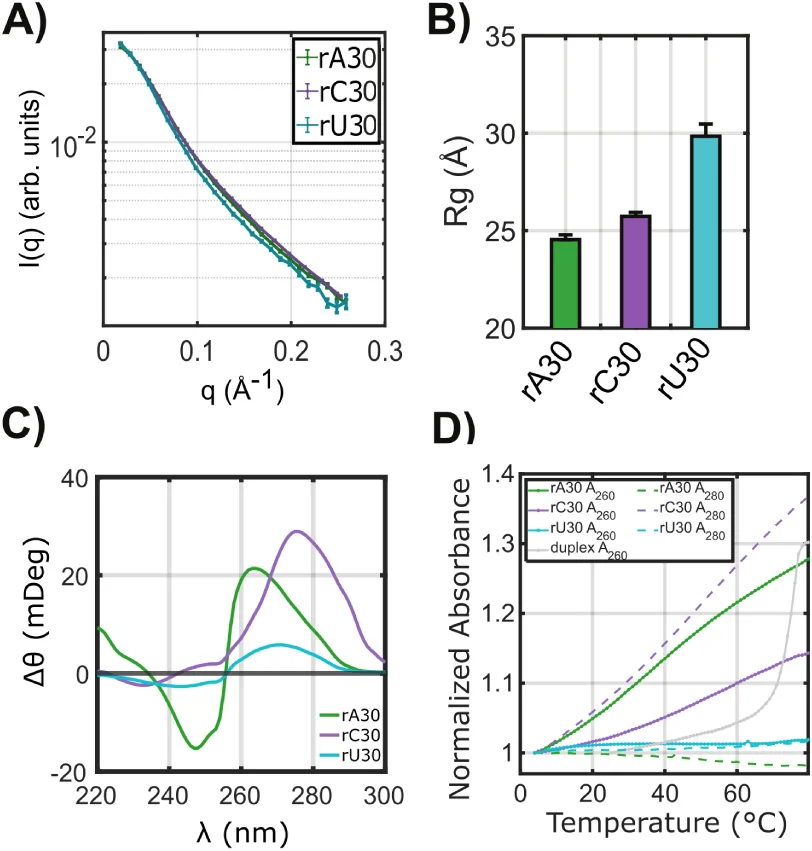
Characterization of rA30, rC30, and rU30 ssRNA by solution measurements. A) SAXS profiles of rA30, rC30, and rU30 under ionic conditions of 100 mM NaCl. Error bars are calculated using BioXTAS RAW.^34^ B) SAXS-derived radii of gyration (*R_g_*) of rA30, rC30, and rU30 in 100 mM NaCl. Error bars represent uncertainties in linear Guinier fits used to obtain *R_g_.* C) Experimental circular dichroism spectra of rA30, rC30, and rU30, illustrating the relative helical character of each ssRNA species. D) UV melting curves of rA30, rC30, and rU30, showing the normalized changes in *A*_260_ and *A*_280_ absorbance as a function of temperature. An RNA duplex standard is shown in gray to illustrate a sharp secondary-structure transition.

In addition to CD, we measured the UV melting curves. Compared with a canonical RNA duplex (gray line), the exhibited transition observed in ssRNAs likely reflects changes in base stacking with increasing temperature. The data suggest that more stacking is present in rA30 and rC30 relative to rU30, as evidenced by an increase in *A*_260_ absorbance as a function of temperature in these two sequences (Figure 1D). The gradual temperature-dependent increase in the melting curves indicates a weak cooperative transition. rU30, on the other hand, does not show a notable change upon heating, suggesting weak stacking/helix content. These results are consistent with earlier studies of these sequences, supporting the idea that poly­(rA) stacks form helical regions. At the same time, poly­(rU) adopts more random coil-like states.^9^ Melting curves measured at 280 nm, where the CD signal of rC30 is higher, further indicate that the stacking/helical structure of poly­(rC) is distinct from that of poly­(rA) (Figure 1D).

### SAXS-Refined MD Simulations Reveal Sequence-Dependent Conformational Ensembles

The experimental data suggest that intrinsic sequence-dependent differences influence the structural propensities of ssRNA strands. The differences observed in melting curves, CD spectra, and SAXS profiles necessitate a more detailed investigation of the conformational ensembles. To gain this insight, we coupled MD simulations with SAXS data using the maximum entropy principle.^26^ This method refines MD-generated sampling to achieve improved agreement with measured scattering profiles.^25^

To assess the accuracy of the simulated and refined ensembles, we first computed the deviation between experiment and simulation-computed SAXS profiles (Figure 2B) quantified by χ^2^ (see Supplemental Methods). The low χ^2^ confirms the success of the methodology. To understand the differences observed in CD spectra, we computed the theoretical CD spectra using DichroCalc^30^ (Figure 2C). Despite the systematic shifts in signal positions relative to the experiments, we achieve reasonable agreement between computed and measured CD spectra, distinguishing the three sequences (Figure 2C vs Figure 1C). To better understand the differences in what is encoded in the CD signals between the calculated and experimental spectra, we generate CD spectra of the rA30 ensemble while varying the transition-energy parameters, *E*_1,2_, in the DichroCalc program (Figure S1). We found that shifts in peak position are controlled primarily by the *E*_1_ parameter, which is associated with excitation energies of stacked bases, see ref 30 for details. This analysis also suggests that the discrepancy between calculated and measured spectra likely arises from limitations of the parameters in forward calculations. To identify what controls peak intensity, we generated fully right-handed and fully left-handed conformations and compared their CD signals with that of the partially helical MD ensemble. The results, shown in Figure S2, demonstrate only a weak dependence of CD intensity on helical content. Overall, our analysis indicates that the relative CD intensities of different ssRNA homopolymers are not a direct quantitative measure of helical content. Instead, they depend on base identity, and stacking geometry.

**Figure 2:**
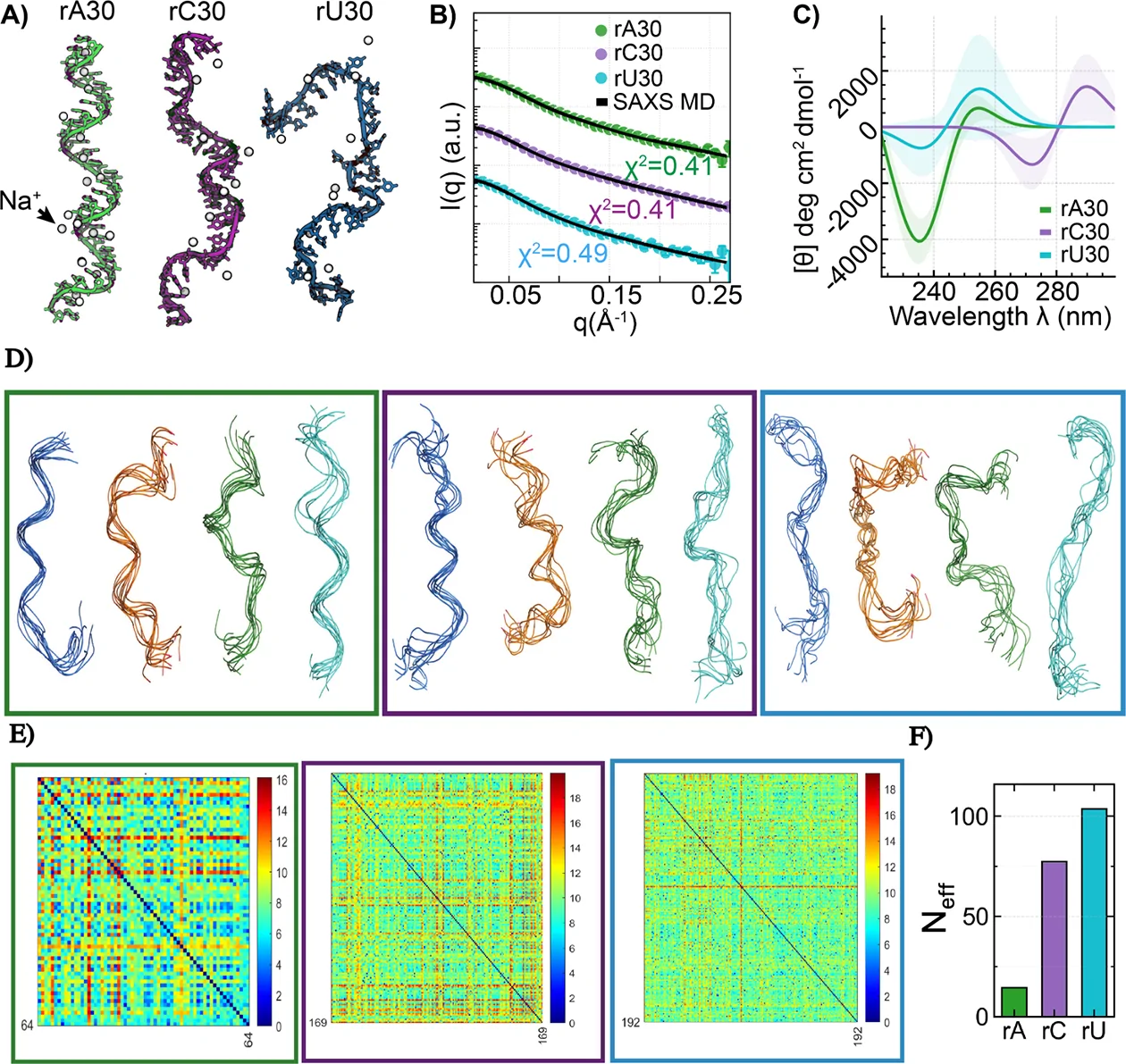
Characterization of experimentally refined conformational ensembles of the sequences. A) The all-atom representation of rA30 (green), rC30 (purple), and rU30 (blue). White circles represent Na^+^ ions. B) Comparison of simulated and experimental SAXS profiles for rA30, rC30, and rU30 in the same salt condition. The agreement between simulation and experiment is quantified using the χ^2^ metric. C) Simulated CD spectra, calculated using the DichroCalc program.^30^ Solid curves represent the ensemble-averaged CD. Shaded regions indicate the range of fluctuations across the ensemble, reflecting structural heterogeneity in the conformational pool. D) 3D clustering of each sampled ensemble in rA30, rC30, and rU30, respectively, a network representation of the structural transitions is shown in Figure S3. E) Pairwise root-mean-square deviation (RMSD) maps showing similarities of each pair of distinct structures in ensembles. The axes limits correspond to the number of conformations in each structural ensemble. F) The effective number of states quantifies structural heterogeneity, *N_eff_*.

Once it was confirmed that the experimental and computed SAXS and CD data reflected sequence dependence effects, we investigated the structures of each sequence in more detail. We began by clustering conformational pools (see Experimental Section). Figure 2D compares the top four populated clusters for each sequence, which together represent the majority of conformations for each molecule. The clusters exhibit clear differences in the degree of helicity among the conformers. In particular, rA30 exhibits a high degree of helicity consistent with earlier study^35^ while more polymer curve variability with greater spatial variance is observed in rC30. The rU30 conformational ensemble, on the other hand, populates coil-like states.

To compare structural ensembles, we clustered the trajectories based on the phosphorus-backbone RMSD using a 6 Å cutoff. We additionally performed clustering including base nitrogen atoms (Figure S4) and observed no substantial changes in rank ordering; therefore, we proceeded with backbone-only RMSD for further analysis. We then constructed pairwise RMSD matrices, which provide the RMSD values between all pairs of these distinct conformers (see Supplemental Methods for details). These values are displayed as a heat map to provide a visual assessment of similarities and differences in each conformational pool (Figure 2E). The RMSD matrices reveal that the structures of rA30 are similar (low pairwise RMSD, blue). In rU30, on the other hand, almost all structures differ by about 8–12 Å. rC30 is also structurally heterogeneous, but its variation is intermediate between the other sequences. We quantify ensemble heterogeneity using the effective number of states, $N_{eff}=e^{-\sum_{i}p_{i}\ln p_{i}}$ where *p*_*i*_ is cluster probability (Figure 2F). The *N*_*eff*_ of rA30 is approximately 10 times lower than that of rU30, which has a similar number of states to rC30. Thus, rC is unique among these sequences, displaying both high heterogeneity and fairly high structural order.

### Base Stacking Propensities Drive Sequence-Dependent Helical Order

In the analysis thus far, we found that rC30 lies intermediate between rU30 and rA30 in its structural ensembles and in the degree of conformational heterogeneity. To gain detailed insight into the basis of these differences, we computed metrics of molecular conformations that are intuitively interpretable and in some cases readily reflected in the data, such as *R*_g_ distribution, stacking propensities, and base orientation correlations (Figure 3).

**Figure 3:**
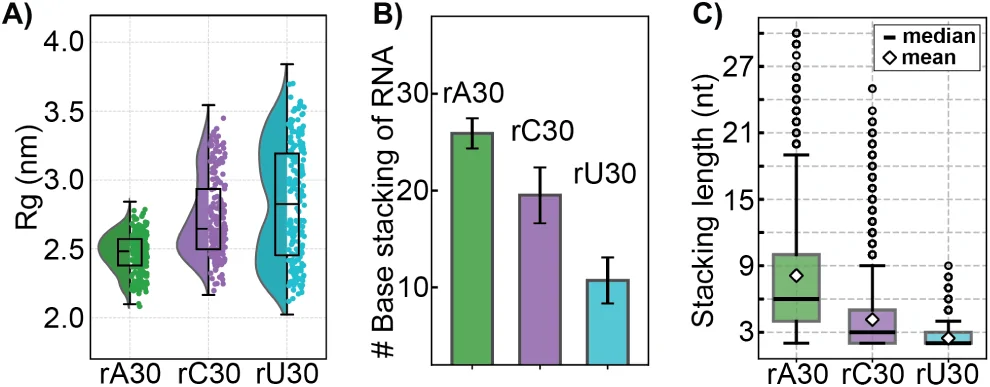
Global and local structural order of the sequences. A) Radius of gyration (*R_g_*) distributions shown as half-violin plots overlaid with box plots. The box indicates the interquartile range, the line denotes the median, and the overall shape conveys ensemble variability. B) Bar chart of average base stacking counts across the RNA sequences, with error bars representing standard deviations. C) Box plots showing the distribution of base stacking segment lengths, with the mean and median values indicated. Black circles indicate outliers beyond the whiskers (points outside 1.5× the interquartile range). Poly­(rA) shows a compact helical structure, while poly­(rU) is reminiscent of a random coil, with poly­(rC) lying between the two. The full distributions of base stacking lengths are provided in Figure S6.

The average *R*_*g*_ computed from the structures in the MD ensembles shows the same trend as the experimental *R*_*g*_ in Figure 1B: molecular size increases from rA30 to rC30 to rU30 (Figure 3A). Surprisingly, simulations indicate bimodal distributions for the *R*_*g*_ of rC30 and rU30, whereas rA30 remains largely unimodal. To understand that better, we compute the *R*_*g*_ of each cluster (Figure S5). rA30 shows similar *R*_*g*_ values across its four clusters (Figure S5A), consistent with the unimodal distribution. For rC30, the clusters with high helical content (Cluster 1–3) show similar *R*_*g*_ to the rA30 while higher-*R*_*g*_ mode arises from the extended state conformations where helical content is less (Cluster 4) (Figure S5B; see also Figure 2D, middle). For rU30, lower-*R*_*g*_ states (clusters 2, 3) are collapsed coil, whereas high *R*_*g*_ clusters (1, 4) are extended coil like structures (Figure S5C; Figure 2D, right). The number of base-stacking interactions and their stacking-length distributions reflect the sequence-dependent degree of structural order. The average number of locally stacked segments (Figure 3B) correlates with the melting behavior shown in Figure 1D. In rA30, about 85% (25/29) of bases are stacked at any given time. In contrast, rU30 shows low stacking propensity ∼34%, and rC30 has ∼68% of bases stacked. RC30 is in between the other two. The stacking-length profiles (Figure 3C) indicate that, on average, bases within rA30 form about 9 consecutive stacked pairs interrupted by occasional base flipping, while stacked regions of rC30 and rU30 consist of about 4–5 and 2 nucleotides, respectively. These numbers correlate with the melting curve results of Figure 1D and are consistent with previous single-molecule force spectroscopy results.^19,20^

To assess the impact of base stacking on overall structural order, we use the orientational correlation function (OCF), which reports the average cosine of the angle between bond vectors of pairs of backbone phosphates as a function of the number of residues that separate them (see Supplemental Methods). The decay length of orientational order and the OCF periodicity are measures of the helical order.^9^ Figure 4A shows the comparison of OCF’s computed for each of the three sequences. The periodicity observed for rA30 is about 8–9 nt, coinciding with the average consecutive base stacking lengths (Figure 3C). The persistence of the correlation suggests that rA30 retains helical order on the 30-nt length. rC30 shows a smaller periodicity (∼6 nt) and a shorter correlation length than rA30. rU30, on the other hand, shows minimal helical order, consistent with a more random coil-like picture.

**Figure 4:**
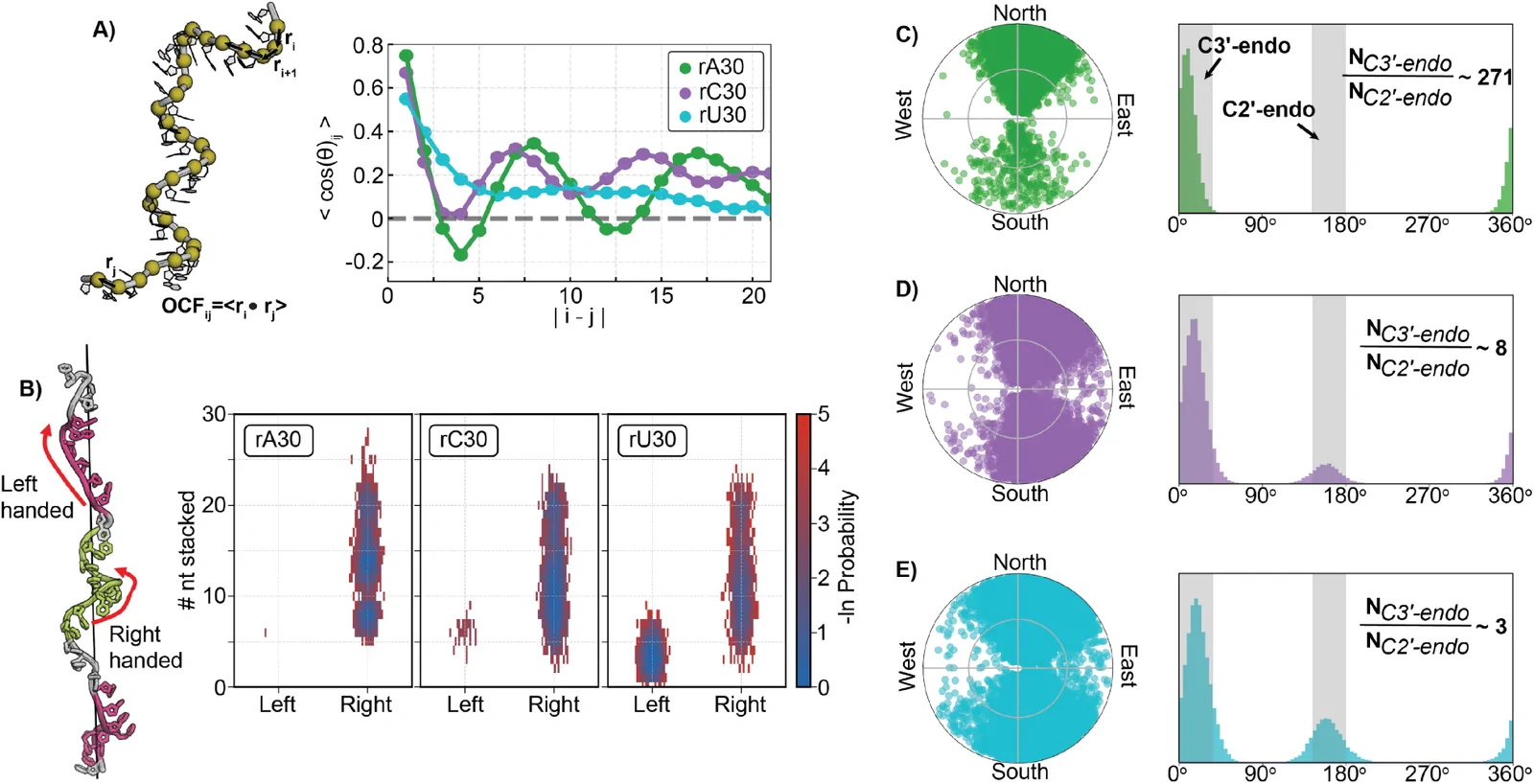
Sequence-dependent helical order and handedness, conformational states of puckering. A) Orientational correlation function (OCF) profiles of ssRNA ensembles reflecting backbone helicity. B) Distributions of the longest handed segment and its base stacking length for each sequence. The *Y*-axis reports the number of consecutively stacked nucleotides within the single longest handed segment, and the *X*-axis reports the handedness of that segment. Long helices are right-handed, with some short left-handed helices appearing in rC30 and rU30. C–E) Phase angle diagrams for sugar pucker; the C2′-endo fraction increases monotonically from rA30 to rC30 to rU30.

To complement the calculations of orientational correlations, we performed an analysis to assess the handedness of the helical stacks (Figure 4B). A clear coupling between local order and helical geometry is evident: long stacked segments are overwhelmingly right-handed, whereas left-handed segments, when present, are confined to short stacks. rA30 shows the strongest enrichment of long, right-handed stacks, rU30 is dominated by short segments with mixed handedness, and rC30 is intermediate. To investigate whether the difference in the CD signal of sequences in Figure 1C and Figure 2C is due to the handedness of the helices, we generated idealized left-handed and right-handed rC30 (Figure S2). The measured CD profile of the rC30 is found to more closely resemble the right-handed ideal helix, suggesting that the right helix is dominant in rC30, consistent with simulation analysis in Figure 4B.

As a final analysis of the MD-generated ensembles, we examined the sugar-pucker populations sampled by each sequence. The puckering state is also sequence dependent (Figure 4C–E). The differences between distributions were quantified using Jensen–Shannon divergence (JSD; see SI for details and Figure S7 for all comparisons). JSD measures the magnitude of separation between two probability distributions, whereas statistical significance was assessed by permutation testing. In this test, the observed JSD was compared with a null distribution generated by randomly reshuffling sequence labels; a comparison was considered significant when the JSD value is bigger than 0.05. The sampled probabilities of *p* < 1.0 × 10^–4^ indicate that all pairwise sequence comparisons are statistically significant. However, the magnitude of the differences varies substantially (Figure S7). The largest separation is observed between rA30 and rU30, followed by rA30 and rC30, whereas rC30 and rU30 show only a small difference. Notably, C2′-endo puckers are present in single-stranded RNAs, even though they are generally disfavored (rather than strictly disallowed) in duplex RNAs because of steric constraints. In rA30, puckers are predominantly North (C3′-endo), consistent with A-form-like geometry. By contrast, the rU30 and rC30 ensembles contain a substantial fraction of South (C2′-endo) puckers, indicative of greater conformational flexibility often associated with B-form-like backbone parameters. While C3′-endo is the dominant puckering state, about 10% of the sugars at a given time are at C2′-endo in rC30 with slightly less population in rU30. Overall, our analysis indicates that rA30 favors A-form-like puckers, rU30 and rC30 samples both A- and B-form-like puckers.

## Conclusions and Future Perspectives

Single-stranded RNA (ssRNA) tracts function as signals in diverse biological processes, yet the mechanisms by which molecular partners recognize and distinguish these signals remain incompletely defined. Increasing evidence indicates that homopolymeric tracts adopt sequence-dependent conformations in solution, leading to their specificity. However, their intrinsic dynamics make it challenging to characterize atomic-level differences. Here, we integrate small-angle X-ray scattering (SAXS) with molecular dynamics to investigate structural ensembles for poly­(rA), poly­(rC), and poly­(rU) under matched buffer conditions. Complementary CD and UV melting measurements indicate distinct helical characteristics, providing cross-validation for the structural differences reported by the simulations. We found that the three resulting ensembles are distinct, supporting the view that single-stranded RNA is not unstructured, but rather forms dynamically changing structures that depend on its sequence.

We used several metrics to analyze and compare the ensembles. Molecular size and shape are characterized by SAXS parameters such as the radius of gyration. Order is characterized through network analysis. Helicity is characterized by analysis of base stacking, segment length distribution, orientational correlation functions (OCFs), and sugar pucker statistics (Figures 3 and 4). Of the three RNAs, poly­(rA) is the most compact and structurally ordered with a high degree of base stacking, resulting in longer helical segments. The sugar puckers in poly­(rA) are predominantly C3′-endo (North), which is typical for A-form double-stranded helices. Poly­(rU), on the other hand, is the most coil-like of the three, displaying much less base stacking and a lack of helical order in the OCF. Interestingly, a broad range of sugar puckers is present, including both C3′ endo and C2′ endo. The latter, South, conformers are associated with B-form double-stranded helices. The lack of large constraints due to base stacking and the wide range of accessible sugar conformations is consistent with the higher conformational flexibility of the poly­(rU) ensemble.

By multiple metrics, including Rg and stacking fraction, poly­(rC) lies quantitatively between poly­(rU) and poly­(rA). Consistent with this characterization, both experimental and computed CD spectra support an intermediate level of helicity for poly­(rC). Notably, despite having helical content approaching that of poly­(rA), poly­(rC) exhibits an order-of-magnitude higher conformational heterogeneity compared to poly­(rA) (Figure 2F). This combination of substantial local helicity with greater diversity may help balance recognition-competent order and conformational plasticity in interactions with poly­(rC)-binding proteins. To further explore previous reports of left-handed helicity,^16,36^ we examined the longest local handed segment in our MD-derived ensembles. Although a small fraction of left-handed segments appears in poly­(rC), these are short; longer segments are overwhelmingly right-handed across all sequences (Figure 4B).

The importance of this work falls along several lines. First, it highlights that the structures of even the most monotypic sequences of RNA are affected by sequence-encoded stacking interactions between nucleotides along the chain, with the structural ensembles displaying distinct characteristics for each base. As outlined in the introduction, single-stranded homopolymeric tracts of poly­(rA), poly­(rC), and poly­(rU) have important biological roles.^1,3–8^ Our experimentally consistent, atomically detailed models help us visualize and quantify the structural properties of homopolymers that may enable understanding their biological roles. We selected 30-nucleotide chains for this study. Although this length facilitates the study of sequence-dependent effects, RNA lengths in nature vary from tens to hundreds of nucleotides, raising the question of whether longer or shorter chains exhibit different folding behaviors or more stable long-range order. This will be explored in a follow-up study.

Second, understanding the sequence-resolved ensembles can provide insight into interactions of nucleic acids with partners ranging from salt ions through macromolecules. The availability of atomically detailed models enables us to investigate the impact of ion-mediated charge compensation and base stacking interactions (to be addressed in a companion study). The structural characteristics of each base also inform how macromolecular partners recognize and interact with their nucleic acid binding partners. For example, enzyme binding affinity can be affected by the sugar conformations of nucleic acids.^37^ The distinctness of poly­(rC) relative to poly­(rU) and poly­(rA) may tune the degree of order in RNA sequences, where a state in between the ordered rigidity of poly­(rA) and the high disorder of poly­(rU) may be achieved.

Third, homopolymers are used as model systems to characterize more complex assemblies, such as biomolecular condensates formed by liquid–liquid phase separation.^38–40^ Quantitative metrics such as *R*_*g*_, stacking fractions/segment lengths, OCF periodicity, handedness, and sugar pucker provide parameters for predictive coarse-grained models of condensate material properties. As we seek to unravel the critical interactions between RNA bases and backbone and their protein partners, it is essential to have a detailed understanding of the intrinsic propensities associated with each sequence.

## Supplementary Material



## Data Availability

The SAXS data have been deposited in the Small Angle Scattering Biological Data Bank (SASBDB) (https://www.sasbdb.org/) with the accession codes: SASDYH4 (rC30), SASDFK9 (rU30), and SASDFB9 (rA30).

## Notes

Supplemental methods for MD simulations; SAXS calculations; SAXS-driven MD; spectral clustering; orientational correlation function analysis; pairwise JSD permutation tests; Supplemental figures; additional supporting tables (PDF)
