# Supplementary material for: The Distinct Structural Propensities of Poly-C, A, and U Single-Stranded RNA
Source: Biomacromolecules. 2026 Aug 5;27(9):5976–83. doi: 10.1021/acs.biomac.6c00716 (PMC13576213; doi:10.1021/acs.biomac.6c00716)
Supplement: Supplementary file 1 [file bm6c00716_si_001.pdf]

Supporting information for The distinct structural propensities of poly-C, A, and U single-stranded RNA

Tong Wang,[†,‡] Weiwei He,[¶,§,‡] Scout Fronhofer,[†] Serdal Kirmizialtin,[*,¶,§] and Lois Pollack[*,†]

†*School of Applied and Engineering Physics, Cornell University, Ithaca, NY 14853, USA*

‡*Equal contribution*

¶*Chemistry Program, Science Division, New York University Abu Dhabi, Abu Dhabi 129188, UAE*

§*Department of Chemistry, New York University, New York, NY 10003, USA*

E-mail: serdal@nyu.edu; lp26@cornell.edu

Supplemental Methods

General MD Simulations set up

The initial A-form RNA single strands of the three sequences were constructed using Nucleic Acid Builder (NAB)[1] and subsequently energy-minimized. The energy-minimized structures were placed in a rectangular simulation box measuring 12.0nm×12.0nm×16.0nm, ensuring at least 4.0nm buffer from the RNA atoms. Periodic boundary conditions (PBC) were applied in all dimensions. The system was solvated with TIP3P water[2] and NBFIX ions.[3] RNA interactions were modeled using the HB-CUFIX force field,[4] which incorporates corrections for hydrogen bonding and base stacking to more accurately reproduce experimentally consistent RNA dynamics. Ions were introduced by randomly replacing water to neutralize the net charge. To mimick the experiments we set the bulk concentration to 100 mM Na^+.

All MD simulations were performed using GROMACS 2018.5.[5] Each solvated system was subjected to a 5000-step steepest descent energy minimization that relieved steric clashes

and resolved unfavorable contacts due to solvation and ion placement. Subsequently, each system underwent a multi-step equilibration procedure prior to production runs. Namely, a 5-ns equilibration was carried out in the NPT ensemble at 300 K and 1 bar, using the V-rescale thermostat[6] and the Parrinello–Rahman barostat,[7] followed by a 2-ns equilibration in the constant temperature and volume (NVT) ensemble using the V-rescale thermostat at 300 K. Soft position restraints (force constant of 1000 kJ/nm^2) were applied to all RNA heavy atoms to preserve the initial geometry while allowing the solvent and ions to equilibrate.

In all simulations, we used the leapfrog integration scheme with a 2-fs time step.[8] Non-bonded interactions, including both van der Waals and short-range electrostatics, were computed with a cutoff of 1.1 nm, and the neighbor list was updated every 40 steps. For long-range electrostatics, we utilized the particle mesh Ewald (PME) method,[9] with a Fourier grid spacing of 1.2Å and fourth-order interpolation. Bond constraints for water molecules were enforced using the SETTLE algorithm,[10] while all other covalent bonds involving hydrogen atoms were constrained using LINCS.[11]

Following equilibration, we conducted 100-ns production runs to sample the conformational landscape of each system and to prepare the structures for the subsequent SAXS-driven MD simulations. Trajectories were recorded every 5 ps. Structural clustering was performed on the ensembles obtained from the brute force MD simulations. This analysis was carried out using the gromos algorithm[12] based on RMSD of heavy atom distances with a cutoff of 10.0Å. The centroid structures from the four most populated clusters were served as replicas of the SAXS driven MD simulations in parallel-replica method.[13]

Computing SAXS from explicit-solvent MD simulations

Theoretical SAXS profiles were computed from MD trajectories using an explicit-solvent scattering approach, as previously described.[14,15] A 10 Å spatial envelope was constructed around RNA molecule that includes RNA, water, and ions atoms (i.e. RNA+ion+water system). Beyond the envelope's isosurface, electron density was assumed to be bulk-like. Due to the inherent flexibility of ssRNA, ensemble fluctuations led to an average solvation shell extending ∼30Å from the RNA surface. To account for buffer scattering, an independent 20-ns MD simulation of 100 mM NaCl solution (i.e., Water+ion system) was conducted in the NVT ensemble.[16] As in the RNA+ion+water system, a 10 Å solvation envelope was applied. The excess SAXS signal from the RNA and its hydration shell was determined by subtracting the scattering profile of the solvent system from that of the solute: $I_{comp}(q) = I_{solute}(q) - I_{solvent}(q)$.

The agreement between experimental and simulated SAXS profiles was quantitatively assessed using the reduced chi-squared (χ^2) metric:

$$\chi^2 = \frac{1}{n_q - 1} \sum_{i=1}^{n_q} \left\{ \frac{[I_{exp}(q_i)] - [I_{comp}(q_i)]}{\sigma_{exp}(q_i)} \right\}^2, \quad (1)$$

where n_q is the number of q points, and $I_{comp}(q_i)$ and $I_{exp}(q_i)$ the computed and experimental intensities, $\sigma_{exp}(q_i)$ is experimental error at q_i, respectively.

SAXS-driven MD simulations

The initial structures for SAXS-driven MD simulations were extracted from the brute-force trajectories described above. Detailed theories of the SAXS-driven simulations are described in Ref.[13,17] Here, we employed a parallel-replica SAXS-driven MD approach using $N = 4$ replicas. Replica simulations were run in parallel to sample RNA conformations and were coupled with a harmonic constraint to the SAXS data. This method ensures consistency with the maximum entropy principle,[18–20], thereby minimizing overfitting to experimental data. During SAXS-driven MD, we employed the stochastic dynamics (SD) integrator.[17,21,22] The center-of-mass (COM) motion of the RNA molecule was removed to maintain its position within the envelope. To ensure proper buffer subtraction, the memory time τ was set to 250 ps. The SAXS biasing term was activated after a 15 ns equilibration via a switch function $\alpha(t)$, allowing $I_{com}(q, \mathbf{R}, t)$ to converge prior to introducing the energetic constraints. Scattering intensities were averaged over 1500 q-vectors, with a coupling constant $k_c = 1.5$. SAXS curves were computed every 5 ps over the course of 4-replica 100 ns simulations (totaling 400 ns) to monitor the conformational search process. Conformations with $\chi^2 < 1.0$ were retained to construct the final structural ensembles.

Spectral clustering for ensemble visualization

To visualize structural ensembles of rU30, rA30, and rC30, we decomposed their conformer pools into a handful of representative subclasses using a graph theoretic approach, described in the following reference.[23] A pairwise $< N_{structures} \times N_{structures}$ matrix of spatial root mean squared deviation (RMSD) values between each pair of structures was calculated and binarized into an adjacency matrix A by applying an RMSD threshold. The degree matrix D was determined from A, and both were used to determine a network graph of each structural ensemble by calculating the Laplacian matrix L:

$$L = D^{-1/2} A D^{-1/2} \tag{2}$$

The RMSD threshold was then iteratively optimized by maximizing the following metric

$$argmax_{R\epsilon(0,20)} \left|\left| \frac{1}{\sqrt{N_{edges}(R)}} < C_c(R) > (1 + 0.1 C_e(R)) \right|\right| \tag{3}$$

which considers the number of edges (N_{edges}), closeness centrality (C_c), and edge connectivity (C_e), and attempts to create a graph without too many connections while containing enough edges for all nodes to be connected. For all conditions in this study, an RMSD threshold of 7 $\mathring{A}$ was found to yield graphs that were well separated.

The optimized network graphs separate RNA conformations that are close together in space. Subclasses were identified from the graphs using spectral clustering.[24] Structures within each subclass are represented by sampling the RNA backbone phosphorus atoms and plotting backbone contours as a wireframe. RNA structures were visualized in ChimeraX v1.8 (UCSF).[25] Full spectral clustering results are shown in Figures S3. Clustering was also performed using the GROMOS algorithm based on pairwise RMSD between conformations,

and is shown in Figure 2D.

Orientation Correlation Function (OCF)

To characterize the structural coherence along the RNA single strands, we computed the orientational correlation function (OCF), defined as the ensemble-averaged dot product of normalized backbone vectors:[26,27]

$$\mathrm{OCF}(|i-j|) = \langle cos\ \theta_{ij} \rangle = \langle \hat{r}_i \cdot \hat{r}_j \rangle \tag{4}$$

Here, $\hat{r}_i$ denotes the unit vector between the ith and $(i+1)^{\mathrm{th}}$ phosphate atoms along the RNA chain. The OCF measures how base-to-base directional correlations decay with increasing residue separation $|i-j|$.

Pairwise comparison of pucker-angle distributions

Pairwise differences between pucker-angle distributions were assessed using Jensen–Shannon divergence (JSD) with a permutation test.[28,29] For each sequence pair, histograms were computed using the same binning as in Fig. 4C–E, pooled, and randomly repartitioned 10,000 times while preserving the original sample sizes to generate a null distribution. For two normalized distributions (P) and (Q), JSD was defined as

$$JSD(P,Q) = \frac{1}{2}D_{KL}(P|M) + \frac{1}{2}D_{KL}(Q|M), \quad M = \frac{1}{2}(P+Q), \tag{5}$$

where D_{KL} is the Kullback–Leibler divergence. Using `scipy.spatial.distance.jensenshannon`,[30] JSD was calculated with the returned Jensen–Shannon distance squared to obtain divergence. The empirical one-sided (p) value was estimated as

$$p = \frac{N_{JSD_{perm} \geq JSD_{obs}}}{N_{perm}}, \tag{6}$$

where $N_{JSD_{perm} \geq JSD_{obs}}$ denotes the number of permuted JSD values greater than or equal to the observed JSD, and $N_{perm} = 10{,}000$. Cases with zero exceedance were reported at the permutation-resolution limit as ($p < 1.0 \times 10^{-4}$). Results are shown in Fig. S7.

Supplemental Figures

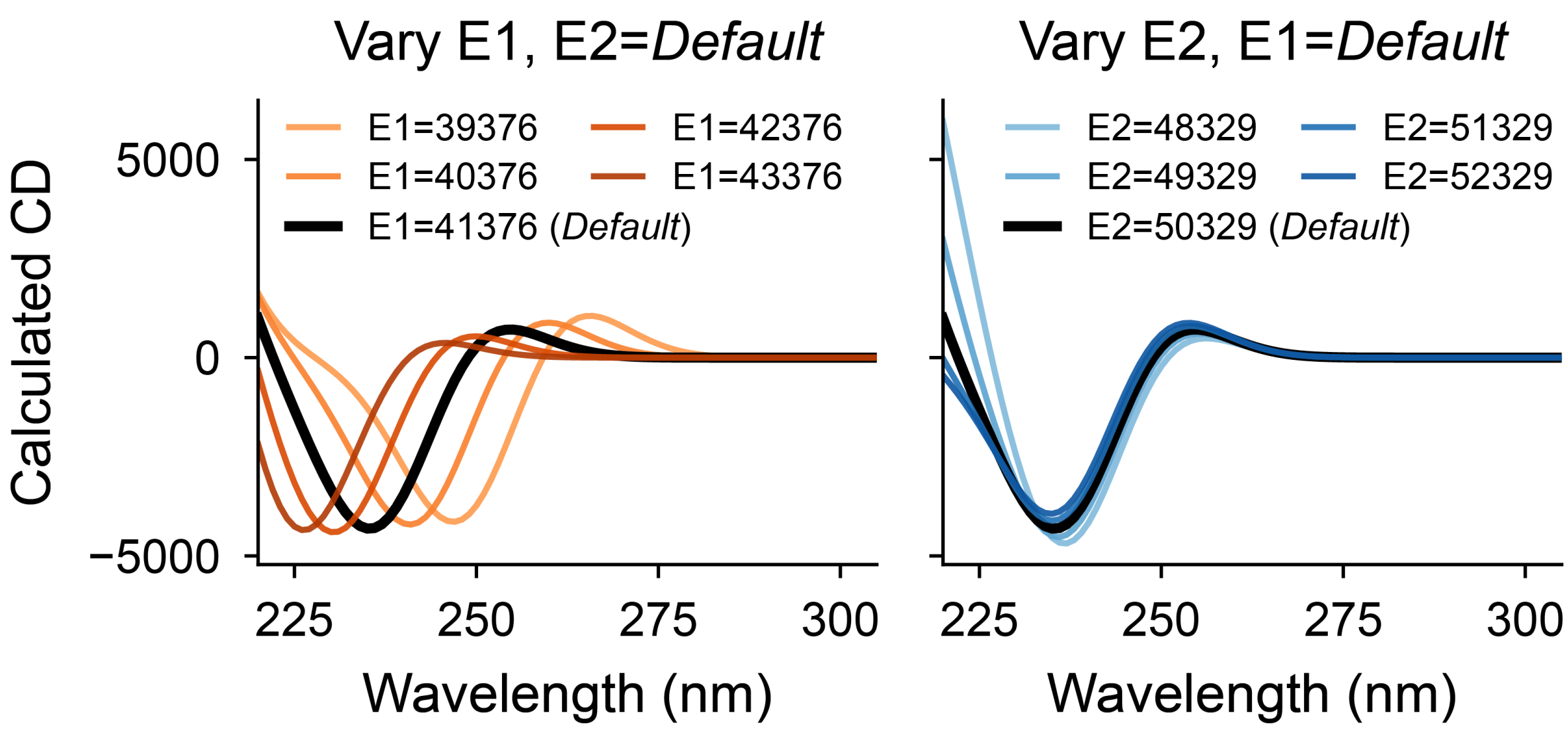

Figure S1: Parameter sensitivity of the DichroCalc CD calculation for rA30. The two local adenine transition-energy parameters, E_1 and E_2 (see Supplemental Tables, Listing 1), in `ADE-FUL3.par` were varied while all other parameters were kept unchanged. The black curve shows the default calculation. Varying E_1 causes pronounced shifts in the calculated CD bands, whereas varying E_2 has a smaller effect over the 220–305 nm range.

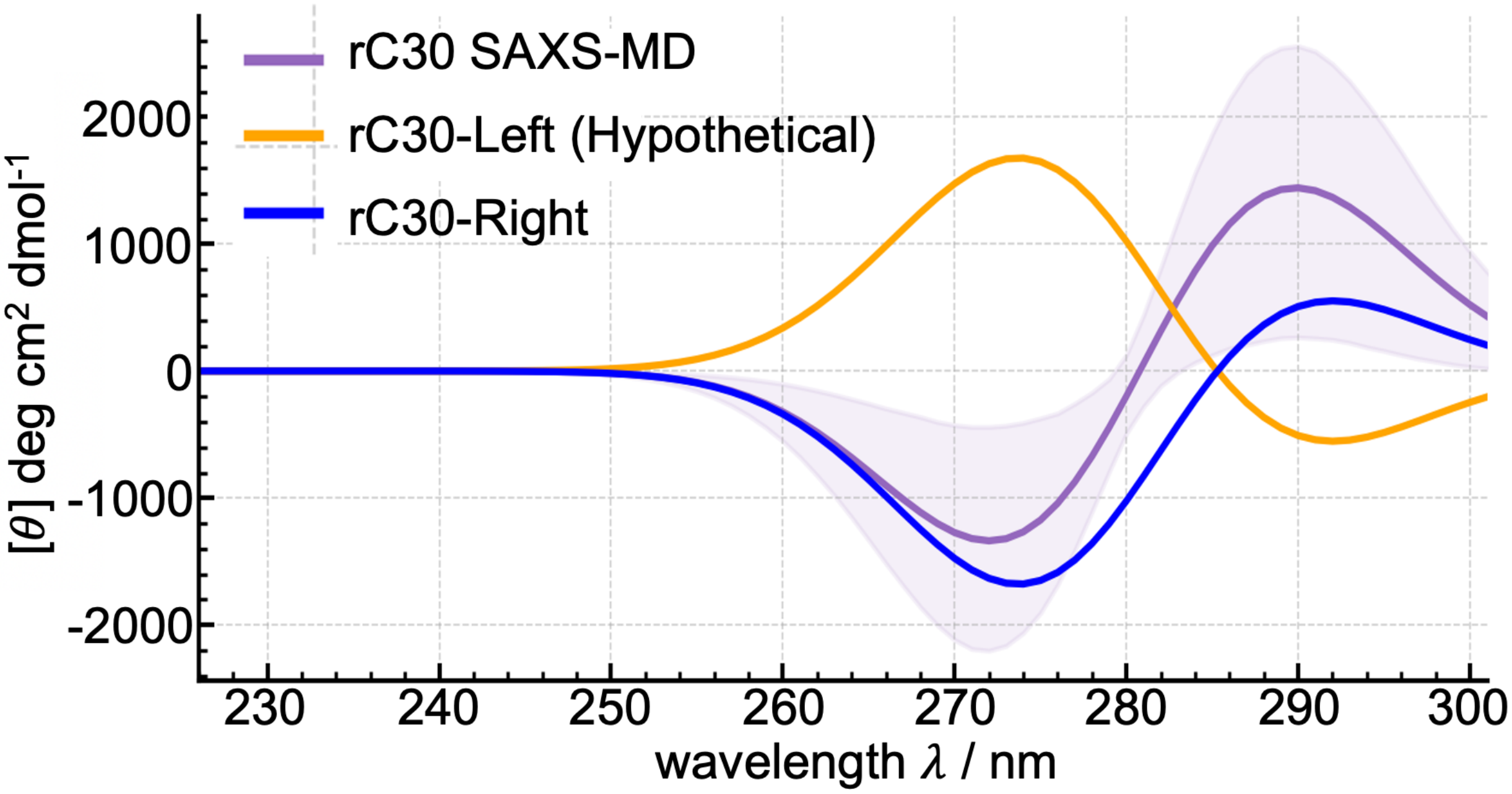

Figure S2: Comparison of poly(rC) circular dichroism signal: SAXS-guided MD ensemble average (rC30 SAXS–MD), and idealized helical references (right-hand, rC30-Right; left-hand, rC30-Left).

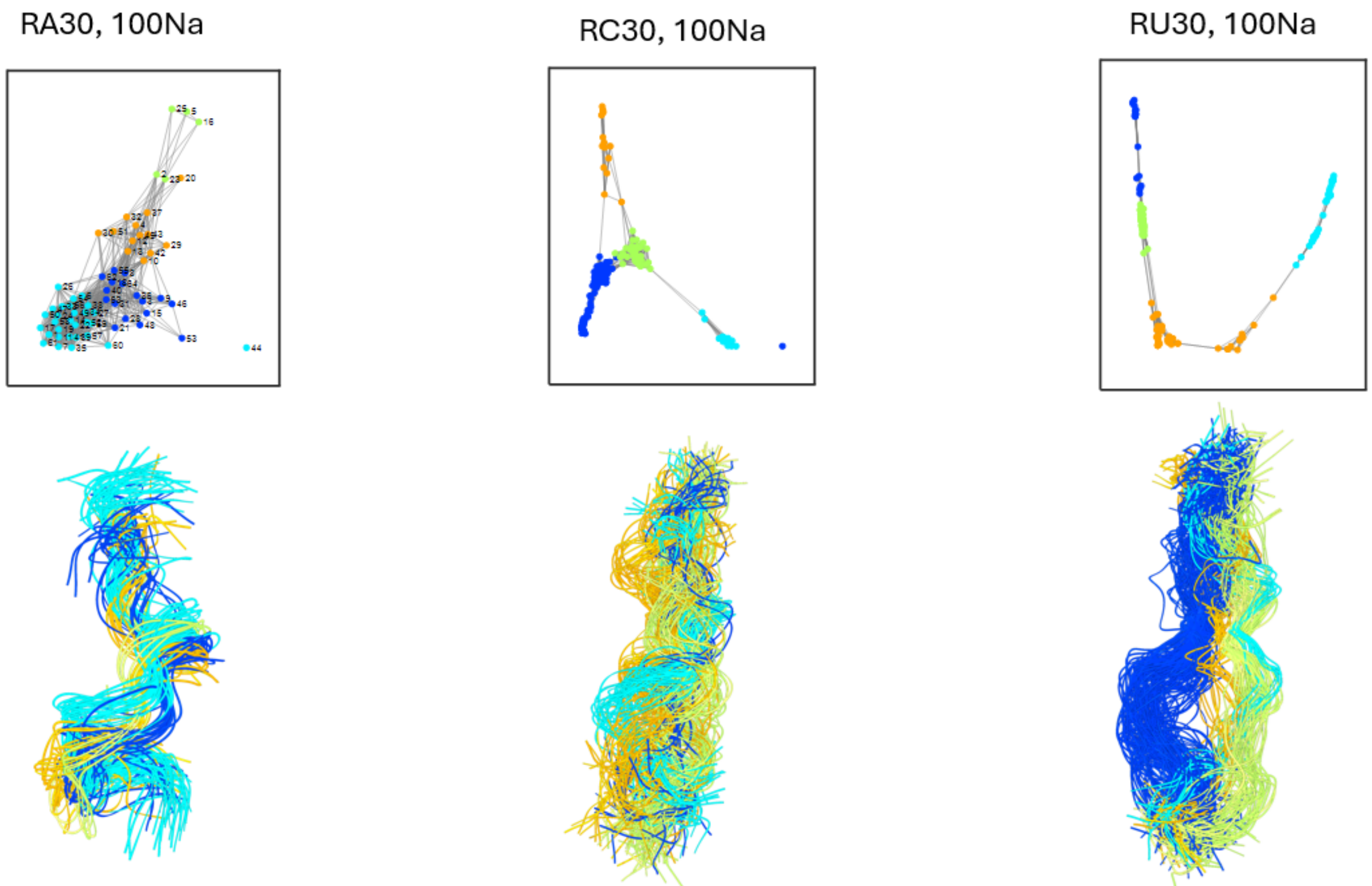

Figure S3: Network graph after RMSD thresholding. Spectral clustering results are shown, where 4 clusters are identified. Backbone conformers are shown as wireframes, colored by cluster identity.

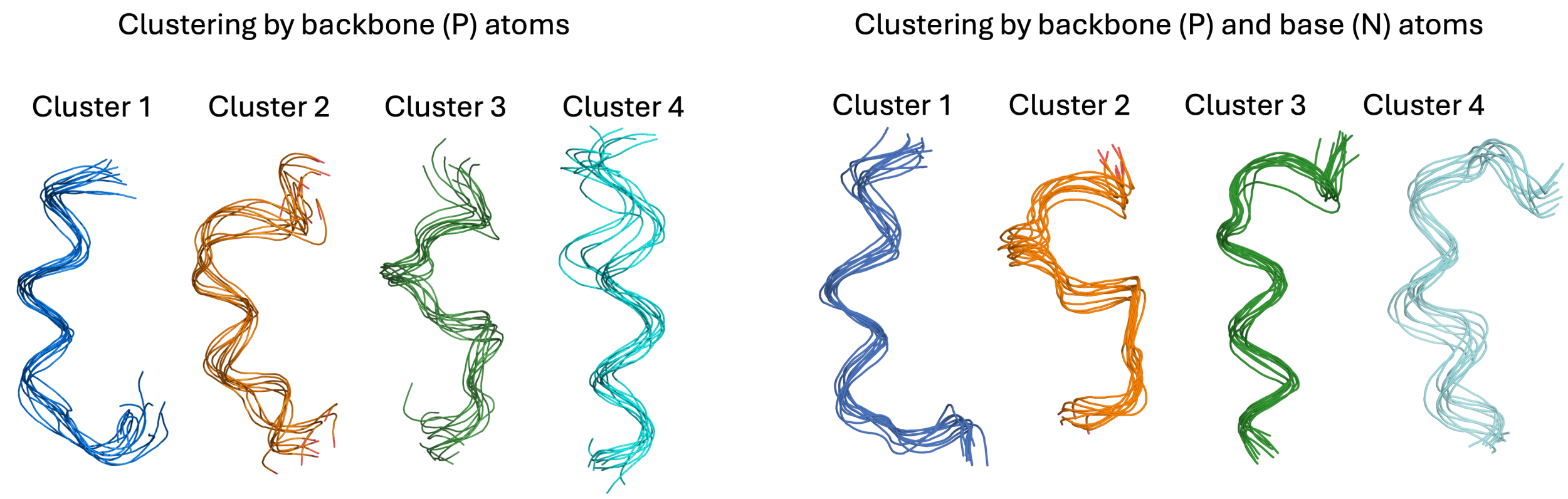

Figure S4: Robustness of clustering to atom selection. RMSD-based clustering was repeated using backbone phosphorus (P) atoms alone and using an expanded atom set that includes base nitrogen (N) atoms, in both cases with a 6 Å cutoff. The dominant conformations are consistent across the two schemes, with differences mainly in cluster rank ordering.

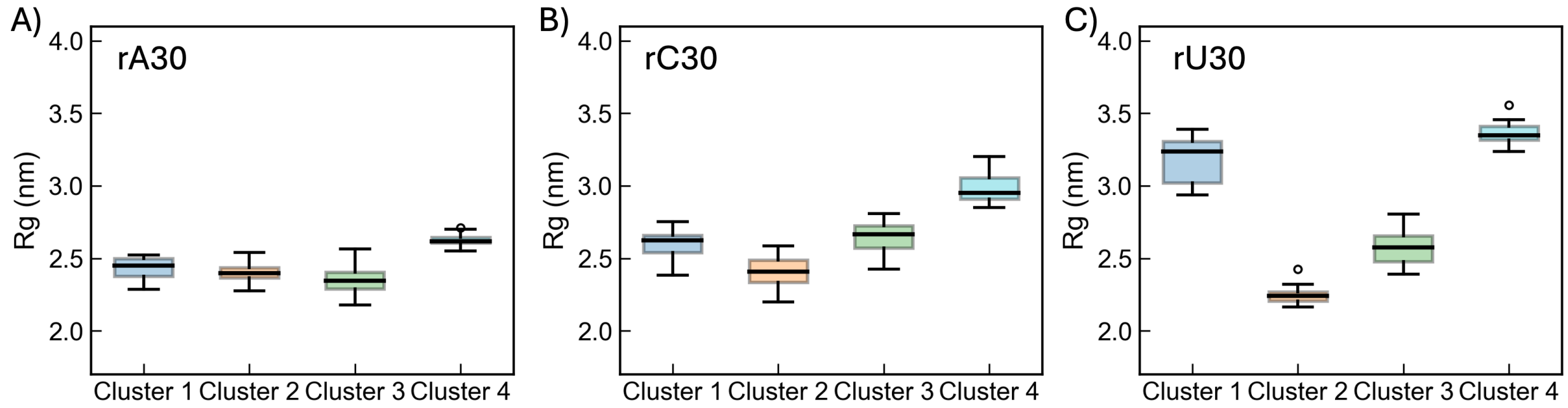

Figure S5: Cluster-resolved distributions of Rg in ssRNA ensembles. Box plots of Rg for the four dominant conformational states identified from clustering of the SAXS-driven MD pools, as shown in Fig. 2D. Boxes indicate the interquartile range, central lines denote the median, whiskers extend to 1.5×IQR, and open circles mark outliers.

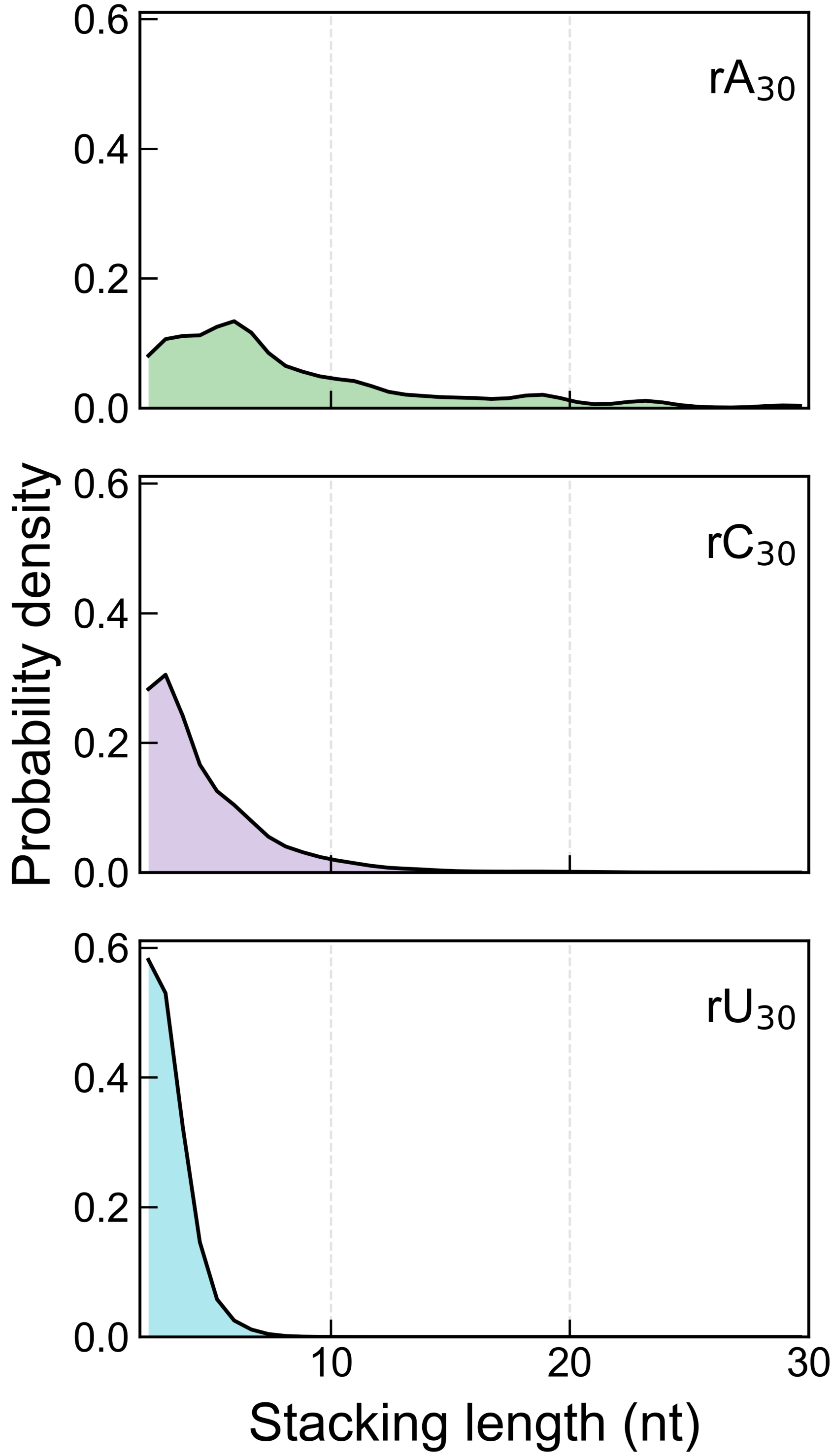

Figure S6: Full distributions of contiguous base-stacking lengths in ssRNA ensembles. Probability density functions of stacking length (nt) for rA30, rC30, and rU30.

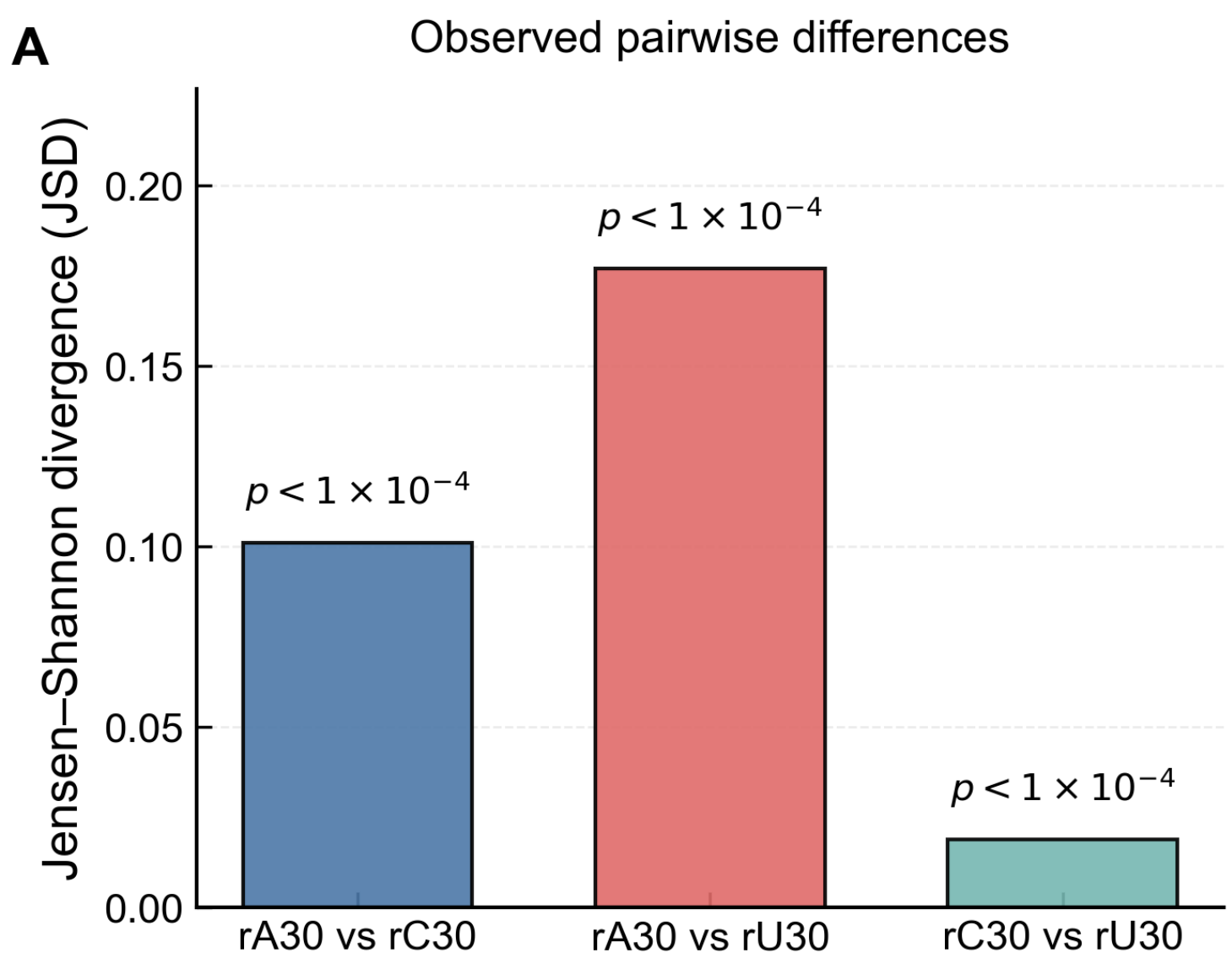

Figure S7: Pairwise permutation analysis of pucker-angle distributions. Pairwise differences among the pucker-angle distributions in Fig. 4C–E were quantified using Jensen–Shannon divergence (JSD). For each sequence pair, histogram counts were pooled and randomly repartitioned 10,000 times while preserving sample sizes to generate the null distribution of JSD values. Gray distributions show the permutation-derived null distributions, and colored diamonds indicate the observed JSD values. All pairwise comparisons were statistically significant: rA30 vs rC30 (JSD=0.1012, $p < 1.0 \times 10^{-4}$), rA30 vs rU30 (JSD=0.1771, $p < 1.0 \times 10^{-4}$), and rC30 vs rU30 (JSD=0.0190, $p < 1.0 \times 10^{-4}$).

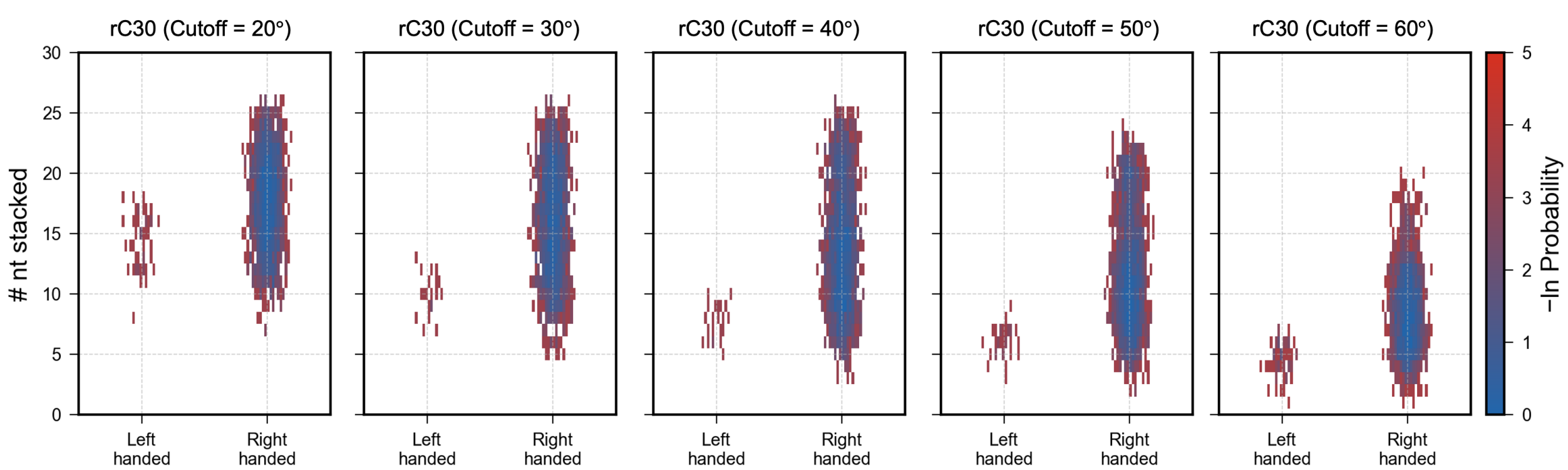

Figure S8: Sensitivity of handedness-stacking analysis to the mean per-step turn cutoff. Distributions of the longest handed segment and its base-stacking length for rC30 were recalculated using cutoff (δ) values from 20° to 60°. The x-axis reports the handedness of the single longest handed segment, and the y-axis denotes the number of consecutively stacked nucleotides within that segment. The cutoff is applied only to handedness assignment, retaining spans with sufficiently resolved phosphate-trace rotation ($|\langle \Delta\theta \rangle| \geq \delta$). Across all tested cutoffs, rC30 remains dominated by right-handed segments. The decrease in stacked counts at higher cutoffs reflects the more stringent selection of shorter, strongly twisted handed segments.

Supplemental Tables

The original adenine (A), cytosine (C), and uracil (U) excitation-parameter files used by DichroCalc CD calculations are shown below.

Listing 1: Original DichroCalc adenine (A) parameter file (`ADE-FUL3.par`). The orange and cyan boxes denote the two local adenine transition-energy parameters, E_1 and E_2, respectively.

```
  -ADE-FUL3-
# only two transitions, one close to 290 nm and the the one with lowest energy around 260nm
# constant moment length of 1 Angstroms
10
        -2.334          0.529          0.000          1.00    # N1
        -2.146         -0.827          0.000          1.00    # C2
        -1.066         -1.501          0.000          1.00    # N3
        -0.000         -0.684          0.000          1.00    # C4
         0.000          0.684          0.000          1.00    # C5
        -1.242          1.308          0.000          1.00    # C6
        -1.390          2.631          0.000          1.00    # N6
         1.278          1.174          0.000          1.00    # N7
         2.053          0.073          0.000          1.00    # C8
         1.342         -1.056          0.000          1.00    # N9
&TRANSITION 1->... # ADE-FUL3
   2    41376.3066         # 241.68 nm            # Transition moment 1.90, angle 23.00 to y axis
      -0.3907      0.9205      0.0000   1.000  # Electr. mom., scale fac.
       0.0000      0.0000      0.0000          # Magnet. mom.
       0.00000000      0.00000000      0.00000000     -1.89677188
      -0.39073113      0.92050485      0.00000000      1.89677188
   2    50329.0747         # 198.69 nm            # Transition moment 6.00, angle -57.00 to y axis
       0.8387      0.5446      0.0000   1.000  # Electr. mom., scale fac.
       0.0000      0.0000      0.0000          # Magnet. mom.
       0.00000000      0.00000000      0.00000000     -5.99649188
       0.83867057      0.54463904      0.00000000      5.99649188
&TRANSITION 2->... # ADE-FUL3
   0     00000.0000     #
         0.0000         0.0000         0.0000  1.000  # electr. mom., scale fac..
         0.0000         0.0000         0.0000         # magnet. mom.
&PERMANENT MOMENTS # ADE-FUL3
   0     00000.0000     #
         0.0000         0.0000         0.0000  1.000  # electr. mom., scale fac..
   0     00000.0000     #
         0.0000         0.0000         0.0000  1.000  # electr. mom., scale fac..
```

Listing 2: Original DichroCalc cytosine (C) parameter file (`CYT-FUL3.par`).

```
  -CYT-FUL3-
# only two transitions, one close to 290 nm and the the one with lowest energy around 260nm
# constant moment length of 1 Angstroms
8
        -0.059         -1.338         -0.005          1.00  # N1
        -1.201         -0.602          0.025          1.00  # C2
        -2.316         -1.186          0.075          1.00  # O2
        -1.194          0.692          0.045          1.00  # N3
         0.003          1.339          0.005          1.00  # C4
         0.000          2.660          0.025          1.00  # N4
         1.234          0.607         -0.045          1.00  # C5
         1.148         -0.718         -0.055          1.00  # C6
&TRANSITION 1->... # CYT-FUL3
   2   35407.7946       # 282.42 nm           # Transition moment 1.91, angle 60.60 to y axis
      -0.8712      0.4909      0.0000   1.000  # Electr. mom., scale fac.
       0.0000      0.0000      0.0000          # Magnet. mom.
       0.00000000      0.00000000      0.00000000     -1.91406826
      -0.87121381      0.49090375      0.00000000      1.91406826
   2   54361.8531       # 183.95 nm           # Transition moment 2.40, angle 14.90 to y axis
      -0.2571      0.9664      0.0000   1.000  # Electr. mom., scale fac.
       0.0000      0.0000      0.0000          # Magnet. mom.
```

```
      0.00000000      0.00000000      0.00000000     -2.39802377
     -0.25713279      0.96637608      0.00000000      2.39802377
&TRANSITION 2->... # CYT-FUL3
   0     00000.0000     #
        0.0000         0.0000         0.0000  1.000  # electr. mom., scale fac..
        0.0000         0.0000         0.0000         # magnet. mom.
&PERMANENT MOMENTS # CYT-FUL3
   0     00000.0000     #
        0.0000         0.0000         0.0000  1.000  # electr. mom., scale fac..
   0     00000.0000     #
        0.0000         0.0000         0.0000  1.000  # electr. mom., scale fac..
```

Listing 3: Original DichroCalc uracil (U) parameter file (`URA-FUL3.par`).

```
  -URA-FUL3-
# only two transitions, one close to 290 nm and the the one with lowest energy around 260nm
# constant moment length of 1 Angstroms
8
       -0.000         -1.396          0.000          1.00  # N1
       -1.366         -0.655          0.023          1.00  # C2
       -2.486         -1.174          0.040          1.00  # O2
       -1.203          0.718          0.021          1.00  # N3
       -0.005          1.397          0.000          1.00  # C4
        0.006          2.625         -0.003          1.00  # O4
        1.151          0.570         -0.022          1.00  # C5
        1.038         -0.785         -0.011          1.00  # C6
&TRANSITION 1->... # URA-FUL3
   2   40327.7842       # 247.97 nm           # Transition moment 3.17, angle -7.00 to y axis
      0.1219      0.9925      0.0000   1.000  # Electr. mom., scale fac.
      0.0000      0.0000      0.0000          # Magnet. mom.
      0.00000000      0.00000000      0.00000000     -3.16531228
      0.12186934      0.99254615      0.00000000      3.16531228
   2   52103.4972       # 191.93 nm           # Transition moment 3.44, angle 23.00 to y axis
     -0.3907      0.9205      0.0000   1.000  # Electr. mom., scale fac.
      0.0000      0.0000      0.0000          # Magnet. mom.
      0.00000000      0.00000000      0.00000000     -3.44038864
     -0.39073113      0.92050485      0.00000000      3.44038864
&TRANSITION 2->... # URA-FUL3
   0     00000.0000     #
        0.0000         0.0000         0.0000  1.000  # electr. mom., scale fac..
        0.0000         0.0000         0.0000         # magnet. mom.
&PERMANENT MOMENTS # URA-FUL3
   0     00000.0000     #
        0.0000         0.0000         0.0000  1.000  # electr. mom., scale fac..
   0     00000.0000     #
        0.0000         0.0000         0.0000  1.000  # electr. mom., scale fac..
```

References

(1) Case, D. A.; Cheatham III, T. E.; Darden, T.; Gohlke, H.; Luo, R.; Merz Jr, K. M.; Onufriev, A.; Simmerling, C.; Wang, B.; Woods, R. J. The Amber biomolecular simulation programs. *J. Comput. Chem.* **2005**, *26*, 1668–1688.

(2) Jorgensen, W. L.; Chandrasekhar, J.; Madura, J. D.; Impey, R. W.; Klein, M. L. Comparison of simple potential functions for simulating liquid water. *J. Chem. Phys.* **1983**, *79*, 926–935.

(3) Yoo, J.; Aksimentiev, A. Improved parametrization of Li+, Na+, K+, and Mg2+ ions for all-atom molecular dynamics simulations of nucleic acid systems. *J. Phys. Chem. Lett.* **2012**, *3*, 45–50.

(4) He, W.; Naleem, N.; Kleiman, D.; Kirmizialtin, S. Refining the RNA Force Field with Small-Angle X-ray Scattering of Helix–Junction–Helix RNA. *J. Phys. Chem. Lett.* **2022**, *13*, 3400–3408.

(5) Hess, B.; Kutzner, C.; Van Der Spoel, D.; Lindahl, E. GROMACS 4: algorithms for highly efficient, load-balanced, and scalable molecular simulation. *J. Chem. Theory Comput.* **2008**, *4*, 435–447.

(6) Bussi, G.; Donadio, D.; Parrinello, M. Canonical sampling through velocity rescaling. *J. Chem. Phys.* **2007**, *126*, 014101.

(7) Parrinello, M.; Rahman, A. Polymorphic transitions in single crystals: A new molecular dynamics method. *J. Appl. Phys.* **1981**, *52*, 7182–7190.

(8) Hockney, R. W.; Goel, S.; Eastwood, J. Quiet high-resolution computer models of a plasma. *J. Comput. Phys.* **1974**, *14*, 148–158.

(9) Darden, T.; York, D.; Pedersen, L. Particle mesh Ewald: An N log (N) method for Ewald sums in large systems. *J. Chem. Phys.* **1993**, *98*, 10089–10092.

(10) Miyamoto, S.; Kollman, P. A. Settle: An analytical version of the SHAKE and RATTLE algorithm for rigid water models. *J. Comput. Chem.* **1992**, *13*, 952–962.

(11) Hess, B.; Bekker, H.; Berendsen, H. J.; Fraaije, J. G. LINCS: a linear constraint solver for molecular simulations. *J. Comput. Chem.* **1997**, *18*, 1463–1472.

(12) Daura, X.; Gademann, K.; Jaun, B.; Seebach, D.; Van Gunsteren, W. F.; Mark, A. E. Peptide folding: when simulation meets experiment. *Angew. Chem. Int. Ed.* **1999**, *38*, 236–240.

(13) Hermann, M. R.; Hub, J. S. SAXS-restrained ensemble simulations of intrinsically disordered proteins with commitment to the principle of maximum entropy. *J. Chem. Theory Comput.* **2019**, *15*, 5103–5115.

(14) Park, S.; Bardhan, J. P.; Roux, B.; Makowski, L. Simulated x-ray scattering of protein solutions using explicit-solvent models. *J. Chem. Phys.* **2009**, *130*, 04B607.

(15) He, W.; Chen, Y.-L.; Pollack, L.; Kirmizialtin, S. The structural plasticity of nucleic acid duplexes revealed by WAXS and MD. *Sci. Adv.* **2021**, *7*, eabf6106.

(16) Wang, T.; He, W.; Pabit, S. A.; Pollack, L.; Kirmizialtin, S. Sequence-dependent conformational preferences of disordered single-stranded RNA. *Cell Rep. Phys. Sci.* **2024**, *5*.

(17) He, W.; Henning-Knechtel, A.; Kirmizialtin, S. Visualizing RNA structures by SAXS-driven MD simulations. *Front. Bioinform.* **2022**, *2*, 2.

(18) Pitera, J. W.; Chodera, J. D. On the use of experimental observations to bias simulated ensembles. *J. Chem. Theory Comput.* **2012**, *8*, 3445–3451.

(19) Roux, B.; Weare, J. On the statistical equivalence of restrained-ensemble simulations with the maximum entropy method. *J. Chem. Phys.* **2013**, *138*, 02B616.

(20) Best, R. B.; Vendruscolo, M. Determination of protein structures consistent with NMR order parameters. *J. Am. Chem. Soc.* **2004**, *126*, 8090–8091.

(21) Chen, P.-c.; Hub, J. S. Interpretation of solution x-ray scattering by explicit-solvent molecular dynamics. *Biophys. J.* **2015**, *108*, 2573–2584.

(22) Chen, Y.-L.; He, W.; Kirmizialtin, S.; Pollack, L. Insights into the structural stability of major groove RNA triplexes by WAXS-guided MD simulations. *Cell Rep. Phys. Sci.* **2022**, *3*, 100971.

(23) Wang, T.; Coshic, K.; Badiee, M.; McDonald, M. R.; Aksimentiev, A.; Pollack, L.; Leung, A. K. L. Cation-induced intramolecular coil-to-globule transition in poly(ADP-ribose). *Nat. Commun.* **2024**, *15*, 7901.

(24) Ng, A.; Jordan, M.; Weiss, Y. On Spectral Clustering: Analysis and an algorithm. *Adv. Neural Inf. Process. Syst.* **2001**, *14*.

(25) Meng, E. C.; Goddard, T. D.; Pettersen, E. F.; Couch, G. S.; Pearson, Z. J.; Morris, J. H.; Ferrin, T. E. UCSF ChimeraX: Tools for structure building and analysis. *Protein Sci.* **2023**, *32*, e4792.

(26) Ullner, M.; Woodward, C. E. Orientational correlation function and persistence lengths of flexible polyelectrolytes. *Macromolecules 35*, 1437–45.

(27) Plumridge, A.; Andresen, K.; Pollack, L. Visualizing Disordered Single-Stranded RNA: Connecting Sequence, Structure, and Electrostatics. *J. Am. Chem. Soc.* **2019**, *142*, 109–119.

(28) Phipson, B.; Smyth, G. K. Permutation P-values should never be zero: calculating exact P-values when permutations are randomly drawn. *arXiv preprint arXiv:1603.05766* **2016**,

(29) Ernst, M. D. Permutation methods: a basis for exact inference. *Stat. Sci.* **2004**, 676–685.

(30) Virtanen, P.; Gommers, R.; Oliphant, T. E.; Haberland, M.; Reddy, T.; Cournapeau, D.; Burovski, E.; Peterson, P.; Weckesser, W.; Bright, J.; others SciPy 1.0: fundamental algorithms for scientific computing in Python. *Nat. Methods* **2020**, *17*, 261–272.
